# Cutaneous Myiasis Caused by *Chrysomya bezziana* Larvae, Mexico

**DOI:** 10.3201/eid1612.100938

**Published:** 2010-12

**Authors:** Raul Romero-Cabello, Leticia Calderón-Romero, José T. Sánchez-Vega, Jorge Tay, Raul Romero-Feregrino

**Affiliations:** Author affiliations: National Autonomous University of Mexico, Mexico City, Mexico (R. Romero-Cabello, L. Calderón-Romero, J.T. Sánchez-Vega, J. Tay, R. Romero-Feregrino);; General Hospital of Mexico, Mexico City (R. Romero-Cabello);; Mexican Institute of Social Security, Mexico City (J.T. Sánchez-Vega)

**Keywords:** Cutaneous myiasis, Chrysomya bezziana, parasite, infection, crater-like ulcer, Mexico, zoonoses, letter

**To the Editor:** We report a case of cutaneous myiasis caused by *Chrysomya bezziana* larvae in a 62-year-old woman who had a complex vascular cutaneous anomaly in her lower right extremity for 8 years. On physical examination, in September 2009, she had a nonlimping walk with pink and painful feet and an ulcerative lesion on the internal surface of the right leg above the internal malleolus. This ulcer was large, clean, without evidence of infection, and had tissue in the process of granulation. Adjacent to the upper edge of this lesion, we observed a second, crater-like ulcer ≈2.5 cm in diameter from which drained an abundant, highly purulent, serohematic material (Figure, panel A).

**Figure:**
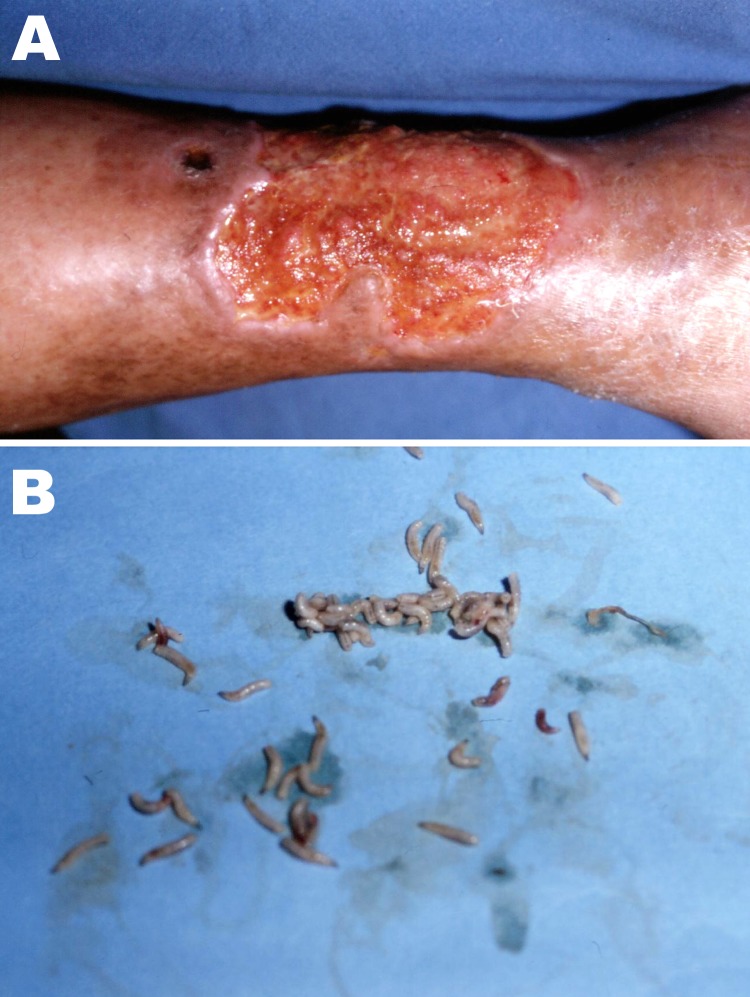
A) Crater-like ulcer ≈2.5 cm in diameter on internal surface of patient’s right leg. B) *Chrysomya bezziana* worms isolated from the ulcer.

Approximately 10 days earlier, the patient had detected discharge of worms from the second lesion, motivating her to seek medical consultation. We performed surgical cleaning and manual removal of worms (Figure, panel B) and referred the patient for external consultation to control vascular, metabolic, and parasitologic evolution and for instruction in proper hygiene. The worms were identified as *C. bezziana* larvae by the Parasitology Laboratory of the Microbiology and Parasitology Department, Faculty of Medicine, National Autonomous University of Mexico.

Myiasis, a zoonotic disease, is defined as invasion of human living tissue by eggs or larvae from flies of the order Diptera. Among the diverse types of human myiasis that can occur in tropical regions, those in skin tissue are the most frequent, especially those generated by flies of the family *Calliphoridae*, of which the predominant species are *Cordylobia anthropophaga* (tumbu fly); *C. bezziana*, and *Oestrus ovis* in Africa (*1*) and *Dermatobia hominis* (American warble fly) in Central and South America.

Myiases have become increasingly relevant, particularly when human activity is carried out in environments with poor hygiene or in close proximity to domestic and peridomestic animals, such as dogs and rats (*2*). Human myiases generally are present in cavities or wounds but also can affect tissue, such as the skin, eyes, oral cavity, intestines, or urogenital area. *C. bezziana* larvae can usually be found infecting wounds or cutaneous ulcers but are occasionally found in normal skin (*3**–**5*).

Tegumentary and exposed-cavity myiases are relatively easy to diagnose because the source larvae can be observed directly. As a result of the taxonomic study of the larvae based on their morphologic characteristics (*6*), we searched the Medline, PubMed, Scielo, and Lilacs databases for articles describing myiasis caused by the identified species. The published literature showed that no prior cases had been documented in Mexico, and only a few cases had been documented in other regions of North America.

Old World flies, such as *C. bezziana* and *O. ovis*, are the most important producers of myiasis from an economic perspective (*7*). The larvae feed on living tissue causing highly traumatic lesions in a great variety of warm-blooded animals. These myiases present a great diversity of clinical profiles, depending on the affected sites. Occasionally, even after elimination of the larvae, they may have subsequent effects, such as septic arthritis or even death, particularly in newborns, older persons, or immunosupressed persons (*8*). The spread of this infection to other countries or even across continents is not yet clear, but an overriding factor is the massive population migration, which poses the risk for introduction of new species at different places in different seasons. No reports were found indicating that this infection could be spread in another form (e.g., by food or water). Another probable cause for spread of this infection is the global change of the weather that helps larvae to survive in places where they could not previously survive (*9*).

In the case presented, the myiasis was limited to localized destruction of the tissue and was not associated with hemorrhagic problems or bacterial infections. However, myiasis can affect deeper structures, including striated muscle and eventually bone, causing severe destruction of these tissues. Considering the potential effects of this disease, timely diagnoses are critical to limit the damage, take appropriate hygiene measures, and if necessary, provide adequate treatment (*10*).
